# Supplementary material for: Ultrasound Elastography to Assess Age of Deep Vein Thrombosis: A Systematic Review
Source: Diagnostics (Basel). 2023 Jun 15;13(12):2075. doi: 10.3390/diagnostics13122075 (PMC10296923; doi:10.3390/diagnostics13122075)
Supplement: Supplementary file 1 [file diagnostics-13-02075-s001.zip › diagnostics-2412652-supplementary.pdf]

Table S1: Search terms and search strategy

**Pubmed**	
**Concept 1: elasticity**	(elast *) [All Fields]
**Concept 2: thrombosis**	(thromb *) [All Fields] OR (clot *) [All Fields]
**Combination of concepts**	(Concept 1) AND (Concept 2)
**Embase**	
**Concept 1: elasticity**	'elast *':ab,ti
**Concept 2: thrombosis**	'thromb*':ab,ti OR 'clot *':ab,ti
**Combination of concepts**	(Concept 1) AND (Concept 2)
**Web of Science**	
**Concept 1: elasticity**	ALL = (elast *)
**Concept 2: thrombosis**	ALL = ((thromb *) OR (clot *))
**Combination of concepts**	(Concept 1) AND (Concept 2)

the asterisk "" is used as search method for truncation; the asterisk "*" represents any group of characters, including no character.*

Table S2. Risk of bias and concerns regarding applicability across QUADAS-2 domains.

**Risk of Bias across QUADAS-2 domains**			
	Low	High	Unclear
Flow and timing	9	0	0
Reference standard	5	4	0
Index test	2	7	0
Patient Selection	5	3	1
**Concerns regarding Applicability across QUADAS-2 domains**			
	Low	High	Unclear
Reference standard	8	1	0
Index test	6	3	0
Patient Selection	6	2	1

Formal evaluation of QUADAS-2

Risk of Bias

Patient selection was found to be at high risk of bias in three studies. In two of them, the risk was related to a case-control-like study design [9,21]. In the third publication, the risk was associated to the possible misclassification of the index thrombotic event, due to the inclusion of patients with recurrent venous thromboembolism [22]. Finally, in one study, the patients' inclusion criteria were not specified [23].
The index test was found to be at high risk of bias in seven out of nine publications, because of prior knowledge of the patient's medical history by the sonographer [9,18,21,23,24,25,26]. Two studies, on the other hand, resulted in a low risk of bias in this domain, thanks to the use of blinding in the evaluation of the elastosonographic outcome [19,22].
Four studies were found to be at risk of bias in the domain concerning the reference standard, due to the classification of thrombosis in acute, sub-acute and chronic with different time cut-offs compared to those foreseen by the definitions of the current review [22,23,25,26].
The domain of the flow and timing of execution of the index test compared to the reference standard resulted in a low risk of bias in all the studies evaluated, since the anamnestic collection necessarily precedes the execution of an instrumental investigation in this clinical context.

Concerns regarding Applicability

The patient selection domain was found to be at high risk of bias in two studies, owing to the inclusion of patients with superficial vein thrombosis [9,23]. In one case [26] the external validity of the patient selection has been defined as unclear, due to the absence of specific definition of the type of venous thrombosis evaluated.
The index test presented a risk related to applicability in three studies which tested SE through evaluation of the colorimetric map [9,22,23]. Studies that have instead evaluated SWE or that have integrated SE with the calculation of the strain ratio present a low risk of applicability concerns related to their higher reproducibility [18,19,21,24,25,26].
In only one case, a high-risk regarding applicability concerns in the domain of the reference standard was detected. In the study by Paluch et al., in fact, the definition of acute and sub-acute thrombosis was related to the temporal distance from the execution of a sclerotherapy treatment, with subsequent non-applicability in different clinical contexts [23].
